# Advances in the structural understanding of opioid allostery

**DOI:** 10.1016/j.tips.2024.12.007

**Published:** 2025-01-17

**Authors:** Nokomis Ramos-Gonzalez, Balazs R. Varga, Susruta Majumdar

**Affiliations:** 1 Department of Anesthesiology and Washington University Pain Center, Washington University School of Medicine, St Louis, MO, USA; 2 Center for Clinical Pharmacology, Washington University School of Medicine, St Louis, MO, USA

## Abstract

Activation of the μ opioid receptor (MOR) can give analgesia, but also has dangerous side effects. Drugs that target MOR through an allosteric site, meaning they bind outside of the usual pocket, present a novel mode of receptor activation with different pharmacology relative to orthosteric drugs. Recent structural studies give valuable new information on how allosteric modulators interact with MOR.

## MOR signaling, use and misuse

The MOR is a Gi/o-coupled **class A G protein-coupled receptor (GPCR)** (see Glossary) found throughout the central nervous system (CNS). When an **agonist** binds MOR, it induces a structural rearrangement that enables interaction with G proteins, leading to their activation. Gα proteins inhibit the accumulation of cyclic AMP (cAMP) whilst Gβγ proteins promote the opening of inward-rectifying potassium channels and the closing of calcium channels. After G protein activation, MOR is phosphorylated by GPCR kinases, which leads to the recruitment of β-arrestins [1]. Activation of MOR gives analgesia, but also causes dependence, tolerance, euphoria, and respiratory depression. Clinically, MOR drugs are widely used to treat pain, but the overprescription of opioids in the USA in the 1990s led to a rise in opioid misuse and a subsequent increase in heroin overdose deaths in the early 2010s. However, since 2013, a steady rise in overdose deaths associated with synthetic opioids like fentanyl has occurred, and in 2022, 82 000 people died from opioid overdose in the USA [2]. Consequently, research into therapeutically modulating MOR with fewer side effects has been proposed as a potential strategy to address the opioid epidemic [3–7].

## Allosteric modulation of MOR: forms and uses

**Figure 1.**
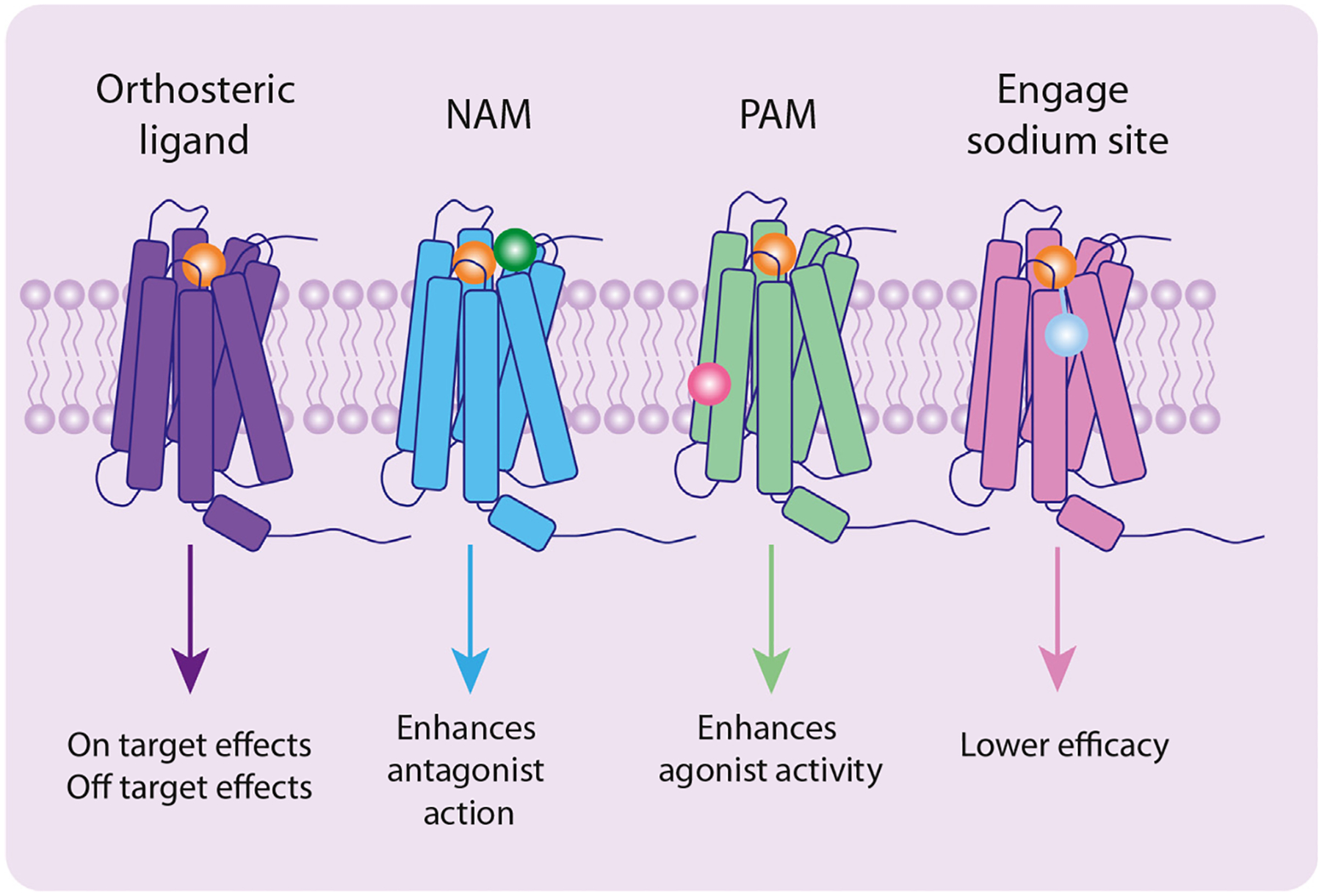
Graphical representation of the effects of orthosteric and allosteric ligands. An orthosteric ligand binds in the orthosteric site and will give on- and off-target effects. A negative allosteric modulator (NAM) will bind to an allosteric site and enhance the activity of an antagonist; it may also negatively modulate agonist activity. A recent study showed that a NAM site exists in the μ opioid receptor (MOR) above the orthosteric binding site [10]. A positive allosteric modulator (PAM) will bind to an allosteric site and enhance the activity of an agonist or will help stabilize the receptor in an active conformation; in a recent study a PAM allosteric site was located at the bottom of transmembrane proteins TM3/4/5 in MOR [9]. Using a bitopic approach, a ligand can sit in the orthosteric site and also bind and activate the allosteric sodium site, leading to decreased potency and efficacy of the parent agonist; a recent study showed that it is possible to bind MOR with a fentanyl biotopic and engage the sodium site either directly [5] or with a water-mediated bridge [4].

Allosteric modulation refers to the binding of a ligand to a site distinct from the **orthosteric binding pocket**. An allosteric ligand can change the activity of an orthosteric ligand or the receptor’s activation state to promote either an active state (positive allosteric modulator, PAM) or an inactive state (negative allosteric modulator, NAM) (Figure 1). Although allosteric ligands generally lack intrinsic activity and rely on the presence of an orthosteric ligand, some modulators may bind but have no effect (silent allosteric modulator, SAM), and in some cases a PAM may have modest intrinsic activity of its own (ago-PAM). An endogenous negative allosteric modulator – sodium ions – also affects MOR. Sodium ions negatively modulate agonist binding to class A GPCRs, including opioid receptors, without significantly altering **antagonist** interactions [8].

Allosteric modulation offers a promising new approach to targeting the MOR with potentially fewer risks than traditional opioid therapies. For example, a PAM could selectively enhance MOR activity, providing effective pain relief while minimizing side effects. This approach could reduce the need for prescribing conventional opioids, which are associated with significant adverse effects, thereby contributing to safer pain management options. Conversely, a NAM that enhances **naloxone** could be particularly useful in opioid overdose scenarios, potentially proving more successful against harmful synthetic opioids such as fentanyl. Currently, no MOR allosteric modulators are in clinical use, and the field is only beginning to explore these opportunities [3–6,9–11]. Until recently, little was known about the specific interactions and binding modes of MOR allosteric ligands. Recent advancements in **cryo-electron microscopy (cryo-EM)** have led to the solving of several MOR structures with allosteric modulators bound, giving novel insights into how these ligands change MOR activation, which may guide future ligand design of MOR modulators.

## Positive allosteric modulation of MOR can be used to enhance the activity of agonists

**Figure 2.**
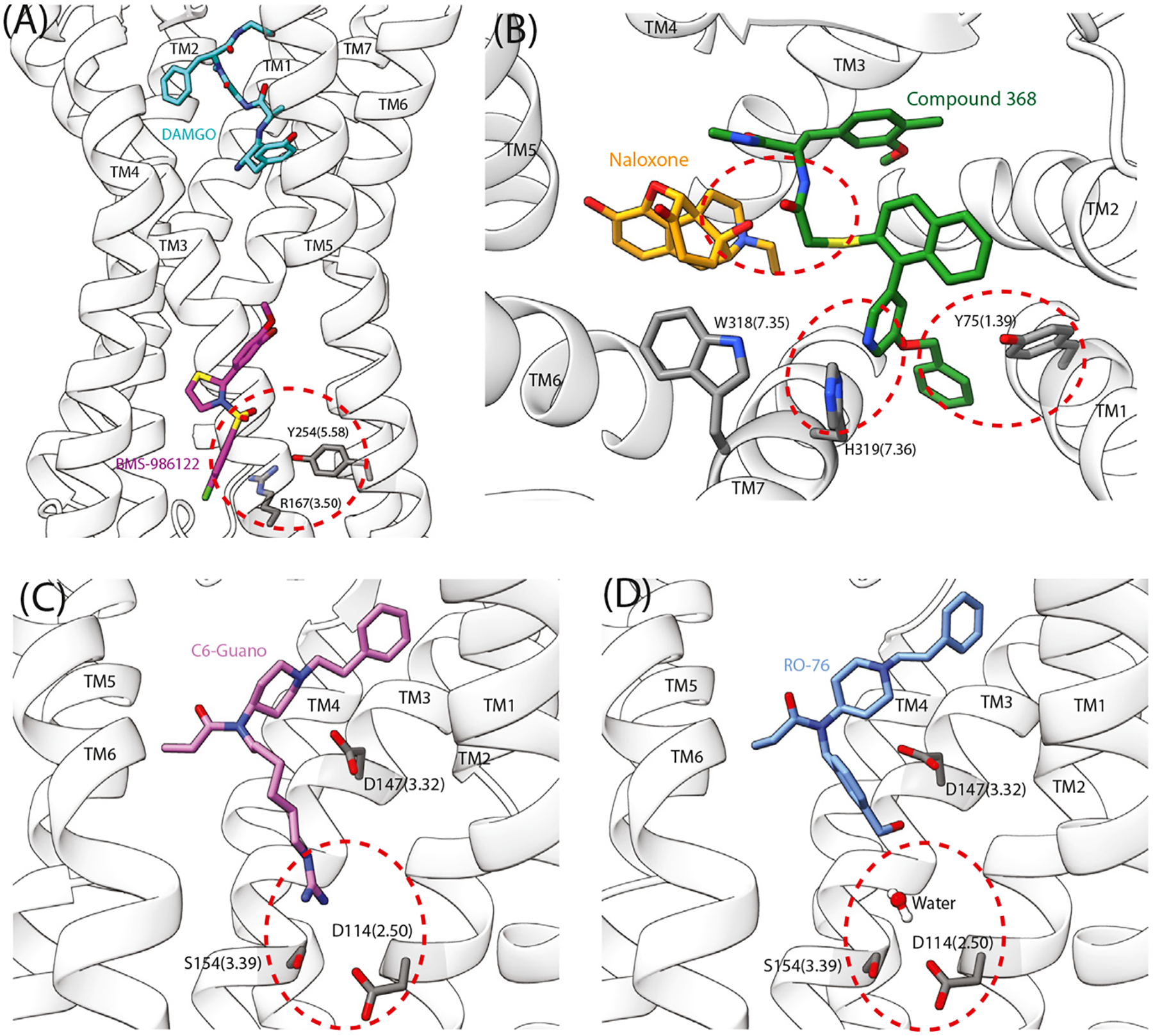
Structures and pharmacology of allosteric ligands. (A) BMS-986122 positive allosteric modulator (PAM) binds to the human μ opioid receptor (MOR) (PDB: 8K9L) between transmembrane proteins TM3 and TM4, and aids in the stabilization of an interaction between R167^3.50^ and Y254^5.58^. DAMGO binds in the orthosteric site. (B) Negative allosteric modulator (NAM) 368 binds in the extracellular vestibule of the mouse MOR with naloxone in the orthosteric pocket (PDB: 9BJK); 368 interacts with Y75^1.39^, naloxone, and H319^7.36^. (C) C6-guano binds to the mouse MOR (PDB: 7U2K) and directly interacts with residues making up the sodium binding site, D114^2.50^ and S154^3.38^. (D) RO76 binds to the mouse MOR (PDB: 9BQJ), a water molecule forms a bridge between the ligand and the allosteric sodium binding site, coordinated by D114^2.50^ and S154^3.38^. In all images ligands are labeled, TMs are labeled, residues of interest are labeled with residue numbers and Ballesteros–Weinstein numbering in brackets, key interactions are highlighted with red dashed circles. In (C) and (D) TM7 has been removed for clarity. Images were made using structures obtained from the Protein Data Bank.

It has been hypothesized that MOR PAMs could be used to achieve analgesia with decreased side effects by enhancing the action of endogenously expressed opioids present in the brain in a pain state. The MOR PAM BMS-986122 improves the potency of endogenous ligands in a range of *in vitro* assays, including inhibition of cAMP accumulation [7], [^35^S]GTPγS binding, and in ^3^ [H]diprenorphine competition binding [12]. *In vivo*, BMS-986122 displayed antinociception without co-administration of an exogenous agonist by enhancing the effect of the endogenous peptides. It also showed fewer side effects relative to morphine [3]. In 2024, a cryo-EM structure of MOR, in complex with Gαiβγ and bound by **DAMGO** in the orthosteric site and BMS-986122 in an allosteric site, was solved [9]. BMS-986122 binds at a lateral pocket of the receptor at the bottom of transmembrane domain 3 (TM3), TM4, and TM5, on the intracellular side of the receptor and positioned outside of the helical bundle, towards the membrane (Figure 2A). The overall receptor conformation was similar with and without BMS-986122, but differences were noted in side chain orientations within the conserved **E/DRY** and **NPxxY** motifs. The combination of the cryo-EM structure and nuclear magnetic resonance (NMR) studies indicated that the presence of BMS-986122 helps to stabilize a hydrogen bond between R167^3.50^ (superscript residue numbers refer to **Ballesteros–Weinstein numbering** [13]), which is part of the E/DRY motif, and Y254^5.58^. This residue in turn forms a hydrogen bond with a key NPxxY motif residue, Y338^7.53^. The structural data indicated that the hydrogen bond observed between R167^3.50^ and Y254^5.58^ may help stabilize an outward swing of TM6 without the presence of G protein, promoting an active conformation [9].

Other work on MOR PAMs led to the discovery of MS1 which acts as a PAM for **endomorphin-1** and L-**methadone** [14], and subsequently Comp5, which showed modest PAM activity in a GoA bioluminescence resonance energy transfer (BRET) assay with methadone and oxycodone [15]. Comp5 enhanced morphine antinociception in a model of postoperative and chronic pain and enhanced the action of oxycodone in a chronic pain model. No cryo-EM structures exist for these PAMs [15].

## Negative allosteric modulation of MOR can be used to increase the activity of antagonists

MOR NAMs can help stabilize the inactive conformation of the receptor. Cannabidiol ((–)-CBD) has been shown to be a MOR NAM: in a recent work several (–)-CBD analogs enhanced naloxone’s ability to reverse fentanyl actions *in vitro* [11], though (–)-CBD has a wide range of targets *in vivo*, including the cannabinoid CB1 receptor.

MOR NAM compound 368 was discovered using a **DNA encoded library** biased towards stabilization of the inactive state by naloxone [10]. Compound 368 was shown to increase the binding affinity and duration of action of ^3^H-naloxone to MOR, and enhanced the potency of naloxone inhibition of agonist G protein signaling in a BRET assay. *In vivo*, 368 enhanced naloxone’s ability to reverse morphine-induced antinociception, respiratory depression, and hyperlocomotion, as well as fentanyl antinociception. A cryo-EM structure was solved with MOR in the inactive state, bound to Nb6, naloxone, and NAM 368 [10]. This revealed that 368 binds in the extracellular vestibule above naloxone and forms a hydrogen bond with the antagonist. NAM 368 increases naloxone’s affinity and potency by stabilizing its binding and hindering its exit from the receptor. It was found to stabilize an outward movement of TM1 by interacting with Y75^1.39^, as well as outward movement of TM7 by interacting with H3197.36 and W3187.35.

## The sodium allosteric site can be engaged to lower the efficacy of MOR agonists

In class A GPCRs a sodium ion can bind in a highly conserved site and help to stabilize the receptor in an inactive state, acting as a NAM. Because the sodium site can be reached from the orthosteric site, a bitopic approach, where both the orthosteric and **sodium allosteric sites** are occupied simultaneously, can be employed [16].

In one study, this bitopic approach was used on the fentanyl backbone by attaching a guanidine moiety via a six-carbon linker to extend into the sodium binding pocket; the resulting ligand was C6 guano [5]. A cryo-EM structure revealed that C6 guano forms a direct interaction with the conserved residue D114^2.50^ in the sodium binding site. Molecular dynamics simulations revealed stable salt-bridge interactions. *In vitro*, C6 guano displayed lower G protein **efficacy** and negligible β-arrestin recruitment compared with its fentanyl backbone. *In vivo*, C6 guano induced potent analgesic effects with fewer side effects than traditional opioids [5].

Following the work on C6 guano, which exhibits poor blood–brain barrier (BBB) permeability, a new bitopic ligand, RO76, was designed [4]. A cryo-EM structure revealed that RO76 interacts indirectly with the conserved D114^2.50^ residue through stable water molecules. RO76 displayed reduced efficacy for the Gi1 pathway and minimal β-arrestin recruitment *in vitro*. *In vivo*, RO76 provided strong MOR-mediated analgesic effects with fewer adverse outcomes compared with morphine, including reduced respiratory depression and physical dependence [4].

## Concluding remarks

Allosteric ligands present a promising avenue for modulating MOR, a key target for analgesics. Recent pharmacological studies and cryo-EM structures have advanced our understanding of how allosteric modulators interact with MOR at a molecular level. Many harmful side effects of MOR agonists come from actions in other parts of the body where MOR is expressed but not necessarily involved in pain signaling. The use of PAMs presents a possible method of giving analgesia with reduced side effects associated with potent MOR agonists; PAMs that enhance endogenous signals will allow the effect to be spatially confined to where the **endogenous opioid** is released [3]. MOR PAM BMS-986122 is a promising lead; however, administration of BMS-986122 to two different strains of mice led to differential effects [3], suggesting that BMS-986122 is effective only in cases where the amount and location of endogenous peptide release is optimal for pain relief. In the case of MS1 and Comp5 [14,15], enhancement of **exogenous opioids** could potentially lower the required dose of drugs like morphine and oxycodone, which reduces their overdose liability. However, their PAM effects seem modest, and their binding conformations are unknown, requiring further research.

Currently, opioid overdose is treated by administering naloxone; however, there is evidence that naloxone may not be fully effective in reversing a fentanyl overdose [17], highlighting the need for better opioid overdose treatments. The MOR NAM, compound 368, can enhance naloxone affinity and prolong its duration of action [10]; this gives it potential as a method for enhancing overdose treatment. However, 368 displayed poor BBB penetration, and its effectiveness in reversing fentanyl respiratory depression has not been described. A more optimal MOR NAM would need improved BBB penetration and would be effective against fentanyl and its deadly analogs.

There is evidence that decreasing the efficacy of MOR ligands may improve their side effect profile [18]; this can be achieved by engaging the allosteric sodium site, either directly [5] or indirectly through water molecules [4]. This sodium site engagement leads to low efficacy agonists, which show promising pharmacology *in vivo*. However, BBB permeability was not optimal in the case of the MOR bitopic ligands; further research using ligands without a charged moiety did improve BBB penetration but the resulting ligand, RO76, still had low potency and efficacy. Future research may focus on refining ligand designs to optimize potency, efficacy, and selectivity.

The recent addition of several cryo-EM structures of allosterically bound or interacting ligands gives a greater understanding of how allosteric sites can be used to modulate receptor conformation and pharmacology. The additional structures will aid in the design of further drugs as, prior to these structures, the precise allosteric sites were not well understood. These structures may lead to structure-based design of further allosteric ligands which at present is a largely unexplored area in the MOR field. As this area of the opioid field develops, we may see the design of more opioid ligands that target allosteric sites, potentially giving rise to new MOR therapies.

## Glossary

Agonist: an agonist binds to a receptor and activates it, causing a biological response.
Antagonist: an antagonist binds to but doesn’t activate the receptor, and can block the action of an agonist.
Ballesteros–Weinstein numbering: a system used to standardize the numbering of amino acids in a G protein-coupled receptor (GPCR) to facilitate comparison between different receptors. The first number refers to the transmembrane domain (TM) and the second number refers to the distance from the most conserved residue in the TM.
Class A GPCR: a GPCR (G protein-coupled receptor) is a seven-transmembrane spanning membrane protein that couples to G proteins to elicit responses in the cell. Class A GPCRs are a subfamily of GPCRs, they are rhodopsin-like receptors and have one orthosteric site within the transmembrane bundle, a sodium allosteric site, and N and C terminal domains.
Cryo-electron microscopy (cryo-EM): an imaging technique in which samples are studied at extremely low temperatures, allowing scientists to visualize 2D and 3D maps of proteins and other biomolecules at near-atomic resolution.
DAMGO: (D-Ala2, NMe-Phe4, Gly-ol5)-enkephalin, a stable analog of the endogenous peptide enkephalin which is highly selective for MOR.
DNA encoded library: a vast collection of small molecules, each attached to a unique DNA sequence, used to rapidly screen for potential drug candidates.
E/DRY motif: a key motif made up of E^6.30^/D^3.49^, R^3.50^, and Y^5.58^, also referred to as the ionic lock, in MOR R^3.50^ interacts with E^6.30^ in the inactive state and Y^5.58^ in the active state, allowing the outward swing of TM6.
Efficacy: the ability of a drug to produce a response in the cell or tissue once it binds to its target receptor.
Endogenous opioid: naturally occurring peptides in the body, such as met-enkephalin
Endomorphin-1: an endogenously expressed peptide highly selective for MOR.
Exogenous opioid: substances originating outside of the body, such as morphine.
Methadone: a MOR agonist that is used to treat opioid use disorder.
Naloxone: naloxone, also referred to as Narcan, is used to reverse an overdose due to its competitive antagonist activity at MOR.
NPxxY motif: a conserved motif made up of N^7.49^, Y^7.53^, Y^5.58^, L^3.43^, and V^6.40^; upon activation, the NPxxY motif rearranges, allowing the hydrogen bonding network in the orthosteric pocket to extend towards the E/DRY motif.
Orthosteric binding pocket: the primary active site on a receptor where endogenous ligands or drugs bind to produce their effect.
Sodium allosteric site: a conserved site in class A GPCRs where a sodium ion binds and helps to stabilize the inactive state.
